# Linking ALDH1 and retinoic acid signaling

**DOI:** 10.18632/oncotarget.26661

**Published:** 2019-02-08

**Authors:** Nicholas O. Markham, Won Jae Huh, Robert J. Coffey

**Affiliations:** Vanderbilt University Medical Center, Nashville, TN, USA

**Keywords:** colon cancer stem cells, ALDH1, retinoic acid, LGR5, differentiation

All-trans retinoic acid (ATRA) is an effective therapy to treat acute promyelocytic leukemia. Its role in colorectal cancer is less well understood, in part, since it has not been fully established which cell types express the retinoic acid receptors. In this issue, Boman and co-workers characterize the population of human colorectal cancer (CRC) cells with elevated aldehyde dehydrogenase (ALDH) activity. Based on limited data, they report that RAR and RXR are expressed in human colonic crypt cells that co-express ALDH1, but not minichromosome maintenance complex component 2 (MCM2). These receptors (especially RXR), ALDH1, and the co-regulatory proteins CYP26A1 and C-terminal binding protein 1 (CtBP1) are all overexpressed in CRC compared to matched normal colonic tissue. Furthermore, HT29 and SW480 cells with high ALDH activity express high levels of RAR and RXR. Addition of the RAR/RXR ligand, ATRA, reduced growth and ALDH activity in these lines and increased expression of the neuroendocrine cell markers NSE and CgA. Liarozole dihydrochloride, an inhibitor of CYP26A1-mediated hydrolysis of ATRA, also reduced HT29 and SW480 cell viability, ALDH activity, and sphere formation in soft agar [1].

ALDH activity has been reported as a marker for normal colonic stem cells and CRC stem cells for nearly a decade [2], but little is known about the fate of these cells and their relationship to colonic stem cells defined by other markers, such as Lrig1 and Lgr5. Cell lineage tracing experiments have not been reported and would be complicated as there are 19 different genes with the potential for ALDH activity. However, it appears that in human CRC the ALDH1A3 isoform is the major contributor to ALDH's enzymatic activity [3].

Key functions of cancer stem cells are tumor initiation and chemoresistance. A subset of CD44^high^ cells with elevated ALDH activity isolated from human tumors have increased tumorigenicity in xenografts over cells with low ALDH activity [4]. Kozovska *et al.* [5] showed that chemical and genetic inhibition of ALDH activity can sensitize HT29 and HCT116 cells to capecitabine and 5-FU chemotherapy. The mechanism(s) for these stem-like behaviors may be related to the production of retinoic acid and its subsequent impact on proliferation-associated target genes or the enzymatic processing and/or detoxification of exogenous aldehyde substrates.

Boman's group previously showed that a subset of ALDH1-positive HT29 and SW480 cells express HOXA4 and HOXA9, and siRNA knockdown of either of these two genes reduced expression of ALDH1, CD166, and Lgr5 [6]. In a separate report, this same group reported that miR23b is increased in CRC cells with high ALDH activity and that this miR reduces Lgr5 expression [7]. ALDH-positive colonic stem cells are much less proliferative than Lgr5 stem cells. In this context, it is of note that induced quiescence of LGR5 cells in organoid culture, achieved by combined blockade of MEK/WNT/NOTCH signaling, results in enteroendocrine cell differentiation [8]. It would be of interest to see if addition of ATRA promoted neuroendocrine differentiation of LGR5 cells in organoid culture.

The study by Boman's team raises the possibility that retinoic acid signaling (and its anti-cancer effect) works at the level of colon cancer stem cells and also shows that retinoic acid signaling may be a promising target to treat CRC in combination with other therapeutics. However, there is much to be done to convincingly show that ALDH1 is a marker for normal and neoplastic colonic stem cells, and to elucidate the detailed mechanisms for how retinoic acid signaling inhibits the progression of CRC. An important advance would be to determine the exclusivity of ALDH activity and Lgr5 as markers for colonic stem cell subpopulations.
